# Cultural adaptation and validation of Japanese medical resident version of the workplace social capital scale: a cross-sectional study

**DOI:** 10.1186/s12909-023-04469-w

**Published:** 2023-06-30

**Authors:** Hirohisa Fujikawa, Daisuke Son, Masato Eto

**Affiliations:** 1 Center for General Medicine Education, School of Medicine, Keio University, 35 Shinanomachi, Shinjuku-ku, Tokyo, 160- 8582 Japan; 2 Department of Medical Education Studies, International Research Center for Medical Education, Graduate School of Medicine, The University of Tokyo, Bunkyo-ku, Tokyo, Japan; 3 Department of Community-based Family Medicine, Faculty of Medicine, Tottori University, Yonago, Tottori Japan

**Keywords:** Workplace social capital, Scale development, Trainee, Medical resident, Burnout

## Abstract

**Background:**

The Workplace Social Capital (WSC) Scale is the most frequently used tool for measuring social capital at work in Western countries. However, there are no corresponding tools for assessing WSC among medical trainees in Japan. Thus, this study was conducted to develop the Japanese medical resident version of the WSC (JMR-WSC) Scale and examine its validity and reliability.

**Methods:**

The Japanese version of the WSC Scale by Odagiri et al. was reviewed and the scale was partially modified for use in the Japanese context of postgraduate medical education. To verify the validity and reliability of the JMR-WSC Scale, a cross-sectional survey was performed in 32 hospitals across Japan. Postgraduate trainees (years 1–6) at the participating hospitals responded to the online questionnaire on a voluntary basis. We tested the structural validity through confirmatory factor analysis. We also examined criterion-related validity and internal consistency reliability of the JMR-WSC Scale.

**Results:**

In all, 289 trainees completed the questionnaire. The results of confirmatory factor analysis supported the JMR-WSC Scale’s structural validity on the same two-factor model as that of the original WSC Scale. Logistic regression analysis showed that, after adjustment for gender and postgraduate years, trainees with good self-rated health had a significantly elevated odds ratio for good WSC. Cronbach’s alpha coefficients showed acceptable internal consistency reliability.

**Conclusions:**

We successfully developed the JMR-WSC Scale and examined its validity and reliability. Our scale could be used to measure social capital in postgraduate medical training settings in Japan to help prevent burnout and reduce patient safety incidents.

**Supplementary Information:**

The online version contains supplementary material available at 10.1186/s12909-023-04469-w.

## Background

Social capital is defined as the resources that individuals and groups have access to through their social networks [1, 2]. Social capital can be conceptualized at both the individual and collective levels. It can be studied at the macro level (i.e., regional or country level), the meso-level (i.e., workplace and community), and the micro level [3]. In recent years, Workplace Social Capital (WSC) has received increased attention because the workplace is considered to be the primary social context to which working-age populations devote the majority of their waking hours [4].

WSC refers to the contextual psychosocial characteristics of the workplace, characterized by interpersonal trust and norms of reciprocity [5]. Previous studies have shown an association between social capital in the workplace and workers’ health. Lower WSC is associated with poorer self-rated health [6], poorer mental health [4, 7–9], and higher mortality [10]. There is mounting evidence that WSC benefits employees. Therefore, it is important to measure employees’ WSC and follow up with them based on the results to promote their health. In contrast, burnout is common and a major concern among medical residents [11–13]. A recent systematic review and meta-analysis revealed that the overall prevalence of burnout was as high as 40% [14]. This finding indicates the existence of a significant problem, as burnout can undermine professionalism, lead to medical errors, reduce the quality of patient care, and lead to various personal consequences (e.g., substance abuse, suicidal ideation, and relationship difficulties) [15–20]. Given its prevalence and the severity of its consequences, immediate action is required to prevent burnout among residents [11]. Although it can be caused by a range of factors, including personal, organizational, and social problems, the precise nature of the work environment may be of great importance [21]. In particular, WSC is crucial, as previous studies have reported the association between higher WSC and lower likelihood of burnout [22–24]. Therefore, a validated scale should be developed to assess WSC among medical residents.

In Western countries, several instruments are available for assessing WSC. Among them, Kouvonen et al.’s WSC Scale is the most frequently used; it was well validated through psychometric analysis in a Finnish Public Sector Study [25].

Odagiri et al. developed the Japanese version of the WSC Scale in 2010 [26]. The scale developed by Odagiri et al. was a translated version of the original English scale developed following a rigorous translation process, including forward-translation and back-translation, followed by a review of the back-translation by the author of the original instrument. However, the Japanese version of the WSC Scale has the following two problems that limit its potential use in a Japanese medical education setting. First, Odagiri et al. used only factory employees. The scale has recently been used in a study of medical settings, but only seven participants out of 440 were doctors, and the study also omitted data on the doctors’ years of postgraduate study [27]. In addition, some of the items in the questionnaire are inconsistent with the Japanese healthcare context. Accordingly, it is unclear whether the Japanese version of the scale can be used for Japanese residents and whether it requires cultural adaptation to the Japanese healthcare setting. Second, the Japanese version of the scale has only been presented at an academic conference in Japan. Publication in an international English-language journal would promote international research on the WSC.

Burnout has become a major problem among medical trainees in Japan [28, 29]. A scale for measuring WSC in this group would make it possible to assess training environments and thereby plan for burnout prevention. Therefore, this study aimed to validate and culturally modify the WSC Scale for use by Japanese medical residents.

## Methods

### Design, setting, and participants

We conducted a multicenter cross-sectional study from July to August 2022. We contacted training directors at 78 postgraduate clinical training hospitals throughout Japan, and 32 agreed to cooperate in this study. Their characteristics are presented in Table 1. We sent survey invitations to medical trainees at the 32 hospitals via email and asked them to complete an online questionnaire using SurveyMonkey. In the invitation email, we informed the trainees that participation was optional, and that nonparticipation would not result in any negative consequences for them. Non-respondents were reminded three times via email to complete the survey.

**Table 1:** Characteristics of the participating hospitals

Characteristics	N (%)
Hospital type	
Community hospital University hospital	2 (6) 30 (94)
Hospital size	
≤ 300 beds 301–600 beds ≥ 601beds	5 (16) 19 (59) 8 (25)
Hospital location	
Hokkaido and Tohoku Kanto Chubu Kinki Chugoku and Shikoku Kyushu	5 (16) 6 (19) 6 (19) 4 (13) 5 (16) 6 (19)

### Measures

The original English WSC Scale, as developed by Kouvonen et al., is an eight-item instrument [25]. According to Oksanen et al.’s study, the factor analysis revealed two-factor structure (vertical trust in the supervisor and horizontal trust in peers) [8]. Odagiri et al. developed the Japanese version of the scale and confirmed its acceptable reliability and validity [26]. The question items are rated on a five-point Likert scale (from 1 = strongly disagree to 5 = strongly agree). Factor analysis revealed the same two-factor structure as that of the original scale: horizontal (Q1–5) and vertical trust (Q6–8) [8, 25, 26]. The average score of the eight items was calculated within a range from 1 to 5 for each item, such that higher scores indicate a higher level of WSC.

In this study, the Japanese version of the WSC Scale was reviewed, and the need for cultural adaptation to the medical training setting in Japan was examined. We decided to modify two of the words in the questionnaire so that they could be used for Japanese residents. First, the term *busho* (“work unit”) is an unnatural Japanese expression for the medical trainee setting. Accordingly, we altered this term to *busho* (*shinryoka* “clinical department”) to make the meaning clear. Second, because only Q5 was written in an interrogative way, the text was changed to a statement. These steps produced the Japanese medical resident version of the WSC Scale (JMR-WSC Scale) (Additional file).

### Statistical analysis

We validated the JMR-WSC Scale by following the three steps.

First, we tested the structural validity of the JMR-WSC Scale by confirmatory factor analysis (CFA), using maximum likelihood estimation. In the CFA, we hypothesized the same factor structure (i.e., a two-factor structure) as that of the original WSC Scale developed by Kouvonen et al. and the Japanese version of the WSC Scale developed by Odagiri et al. [8, 25, 26]. The cut-off value for factor loadings was set to 0.40. We assessed the model fitness using the following multiple criteria: chi-square to degrees of freedom ratio (χ^2^/df) < 5, the comparative fit index (CFI) > 0.95, the root mean square error of approximation (RMSEA) < 0.10, and the standardized root mean square residual (SRMR) < 0.08 [30, 31].

Second, as shown by Kouvonen et al. and Odagiri et al., the measure of self-rated health was used for examining criterion-related validity [25, 26]. Self-rated health was assessed by the following item: “How would you estimate your current state of health?” [32] This item was rated on a 5-point Likert scale: 1 = poor, 2 = fair, 3 = good, 4 = very good, and 5 = excellent. Those who answered 4–5 to this item were classified as the “good” self-rated health group, whereas those who answered 1–3 were classified as the “poor” self-rated health group. JMR-WSC Scale scores were divided into two groups by median value. Referring to previous validation studies, we used logistic regression analysis to calculate the gender and postgraduate years adjusted odds ratio and its 95% confidence intervals for the association between WSC and self-rated health.

Third, we checked Cronbach’s alpha coefficients to examine the internal consistency reliability. Alpha values greater than 0.70 are acceptable [33].

Fourth, we conducted descriptive statistics (e.g., mean and standard deviations). In the present study, all statistical analysis was performed using R version 4.2.1. We used the lavaan package version 0.6–12 [34], semPlot package version 1.1.6 [35], stats version 4.3.0, ltm version 1.2.0 [36], and psych version 2.2.9 [37].

### Ethical considerations

All participants provided their individual informed consent through the survey form. Participants were enrolled in a drawing for one of ten ¥5,000 gift cards. The study received ethical approval from the Institutional Review Board of the University of Tokyo (2022062NI).

## Results

**Fig. 1:**
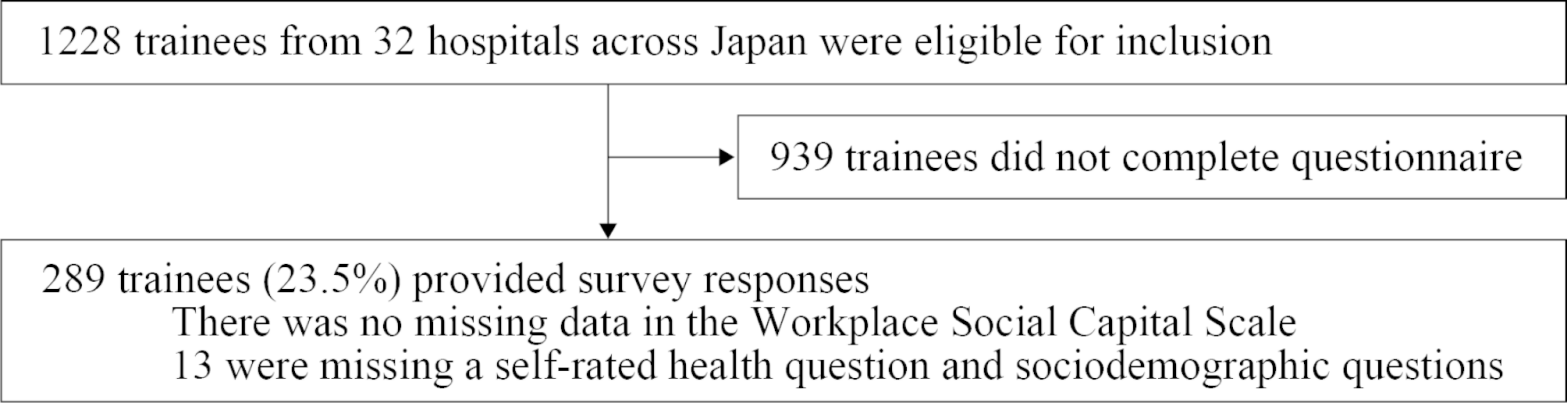
Participants’ flowchart in a study on the cultural adaptation and validation of Japanese medical resident version of the Workplace Social Capital Scale

In all, 289 (23.5%) of the 1228 individuals who were residents of the participating hospitals completed the online survey. The participant selection flowchart is displayed in Fig. 1. Due to the tiny amount of missing data, we chose a complete case analysis. Table 2 provides a summary of the respondents’ characteristics. The replies of the participants to each of the questionnaire items are shown in Table 3.

**Table 2:** Characteristics of the participants (N = 289)

Characteristics	N (%)
Gender	
Female Male Other Data missing	99 (34.3) 176 (60.9) 1 (0.3) 13 (4.5)
Postgraduate years	
1 2 3 4 5 6 Data missing	83 (28.7) 94 (32.5) 46 (15.9) 16 (5.5) 23 (8.0) 14 (4.8) 13 (4.5)
Marriage status	
Unmarried Married Divorced or widowed Data missing	215 (74.4) 57 (19.7) 4 (1.4) 13 (4.5)
Hospital location	
Hokkaido and Tohoku Kanto Chubu Kinki Chugoku and Shikoku Kyushu Data missing	17 (5.9) 143 (49.5) 37 (12.8) 11 (3.8) 42 (14.5) 26 (9.0) 13 (4.5)
Department	
Internal medicine Emergency medicine Ophthalmology Pediatrics Surgery General medicine Otorhinolaryngology Anesthesiology Obstetrics and gynecology Orthopedics Psychiatry Neurosurgery Urology Dermatology Radiology Plastic surgery Data missing	131 (45.3) 16 (5.5) 16 (5.5) 15 (5.2) 15 (5.2) 14 (4.8) 10 (3.5) 9 (3.1) 9 (3.1) 9 (3.1) 8 (2.8) 6 (2.1) 6 (2.1) 5 (1.7) 5 (1.7) 2 (0.7) 13 (4.5)

**Table 3:** Participants’ responses to the Japanese medical resident version of the Workplace Social Capital Scale (N = 289): N (%)

Items (as in original English version)	1 = Totally disagree	2	3	4	5 = Totally agree
Q1. People keep each other informed about work-related issues in the work unit.	1 (0.3)	14 (4.8)	19 (6.6)	146 (50.5)	109 (37.7)
Q2. We have a “we are together” attitude.	4 (1.4)	22 (7.6)	24 (8.3)	134 (46.4)	105 (36.3)
Q3. People feel understood and accepted by each other.	8 (2.8)	18 (6.2)	45 (15.6)	143 (49.4)	75 (26.0)
Q4. People in the work unit cooperate in order to help develop and apply new ideas.	7 (2.4)	22 (7.6)	54 (18.7)	144 (49.8)	62 (21.5)
Q5. Do members of the work unit build on each other’s ideas in order to achieve the best possible outcome?	8 (2.8)	21 (7.3)	45 (15.6)	138 (47.8)	77 (26.6)
Q6. Our supervisor treats us with kindness and consideration.	6 (2.1)	14 (4.8)	22 (7.6)	122 (42.2)	125 (43.3)
Q7. Our supervisor shows concern for our rights as an employee.	8 (2.8)	14 (4.8)	31 (10.7)	122 (42.2)	114 (39.4)
Q8. We can trust our supervisor.	6 (2.1)	10 (3.5)	26 (9.0)	107 (37.0)	140 (48.4)

**Fig. 2:**
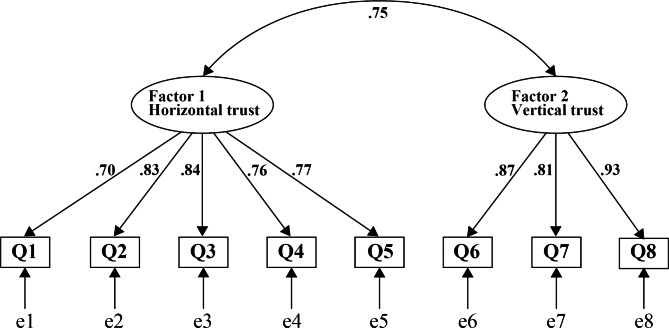
The final confirmatory factor analysis model of the Japanese medical resident version of the Workplace Social Capital Scale. Ellipses represent latent variables (factors). Rectangles are observed variables (items). Values on single-headed arrows are standardized factor loadings. Values on double-headed arrows represent correlation coefficients

To verify the structural validity, we conducted CFA. The path diagrams of the CFA are shown in Fig. 2. All factor loadings exceeded the 0.40 criteria (ranging from 0.70 to 0.93). The model fitness met the recommended criteria: χ^2^/df = 65.707/19 = 3.46, CFI 0.970, RMSEA 0.092, and SRMR 0.028.

We examined the criterion-related validity in relation to self-rated good health. In the logistic regression analysis, trainees with good self-rated health had a significantly elevated odds ratio of 1.83 (1.11–3.04) (p < 0.05) for good WSC.

Table 4 shows internal consistency reliability and score distribution and of the JMR-WSC Scale. We obtained a Cronbach’s alpha value of 0.91 for all items, 0.89 for Factor 1 (horizontal trust), and that of 0.90 for Factor 2 (vertical trust). Thus, we obtained the final version of the scale.

**Table 4:** Internal consistency reliability and score distribution of the Japanese medical resident version of the WSC Scale

	Number of items	Mean	Standard deviation	Observed range	Cronbach’s alpha
Factor 1	5	3.97	0.77	1.00–5.00	0.89
Factor 2	3	4.19	0.86	1.00–5.00	0.90
All	8	4.05	0.73	1.00–5.00	0.91

## Discussion

We developed the JMR-WSC Scale. In a multicenter survey, psychometric analysis indicated acceptable reliability and validity. To the best of our knowledge, the JMR-WSC Scale is the first validated measure that enables us to assess the WSC of medical trainees in Japanese hospital settings.

In our study, the JMR-WSC Scale exhibited a high Cronbach’s alpha value (0.91), indicating a good level of consistency. This finding is consistent with previous studies. In Kouvonen et al.’s study, the sample consisted of workers in Finland, and the alpha value of the WSC Scale was 0.88 [25]. Odagiri et al. tested the scale’s internal consistency reliability in a study of factory employees in Japan and found a Cronbach’s alpha value of 0.90 [26]. Thus, the WSC Scale would be a very useful instrument with good internal consistency across countries and occupations.

The results of the present study confirmed that the JMR-WSC Scale has the same two-factor structure as the original WSC Scale and the Japanese version of the WSC Scale: horizontal and vertical WSC [8, 25, 26]. The horizontal component refers to coworker trust and reciprocity, whereas the vertical component refers to employees’ relationships with their supervisors [8, 38, 39]. Few studies have empirically compared the impact of these different dimensions of WSC on outcomes (e.g., well-being and health). Oksanen et al. conducted a unique study in which they separately analyzed the association between the horizontal and vertical components of WSC and new-onset depression in Finnish public sector employees and identified the importance of both components of WSC as predictors of depression in workers [8]. However, the WSC was influenced by the organization’s and country’s prevailing norms and cultures [40]. Future studies examining the impact of the two components of WSC on various outcomes in various settings, while taking cultural differences into account, would deepen our knowledge of WSC.

The instrument developed in our study can be used to measure social capital in the postgraduate medical training environments in Japan and may help prevent burnout. Since medical trainees’ burnout is currently a serious problem in Japan [41], our tool would be very relevant. Considering that previous studies have suggested a relationship between physician burnout and patient safety incidents [41, 42], our tool could also improve patient care. Furthermore, future development of other versions in other languages would be very appreciated as it will aid WSC research.

Finally, we should note some limitations of the present study. First, we have not examined other psychometric properties (e.g., convergent validity, discriminant validity, and test-retest reliability) beyond structural validity, criterion-related validity, and internal consistency reliability. In future studies, these psychometric properties should be evaluated. Second, the response rate to the questionnaire was relatively small. Online surveys frequently have response rates as low as 10% since it is challenging to have a high response rate [43], and it is not uncommon for the response rate to reach as low as 10% [44]. Referring to recent research findings [45, 46], we believe that our survey’s sample size and response rate are sufficient to provide reliable data.

## Conclusions

We developed the JMR-WSC Scale and then verified its structural validity, criterion-related validity, and internal consistency reliability. The instrument would be useful in evaluating the social capital of medical trainees in postgraduate medical education in Japan, which would lead to preventing burnout and patient safety incidents.

## Electronic supplementary material

Below is the link to the electronic supplementary material.

Supplementary Material 1

## Abbreviations

CFA: Confirmatory factor analysis
CFI: Comparative fit index
JMR-WSC Scale: Japanese medical resident version of the Workplace Social Capital Scale
RMSEA: Root mean square error of approximation
SRMR: Standardized root mean square residual
WSC: Workplace social capital
