# Supplementary material for: Cultural adaptation and validation of Japanese medical resident version of the workplace social capital scale: a cross-sectional study
Source: BMC Med Educ. 2023 Jun 30;23:487. doi: 10.1186/s12909-023-04469-w (PMC10311845; doi:10.1186/s12909-023-04469-w)
Supplement: Supplementary file 1 — Supplementary Material 1 [file 12909_2023_4469_MOESM1_ESM.docx]

The Japanese medical resident version of the Workplace Social Capital Scale (JMR-WSC Scale)

| No. | English version [25] | Japanese version by Odagiri et al. [26] | JMR-WSC Scale |
| --- | --- | --- | --- |
| 1 | People keep each other informed about work-related issues in the work unit. | 仕事に関連した事柄や問題について部署内で情報を共有している | 仕事に関連した事柄や問題について部署（診療科）内で情報を共有している |
| 2 | We have a “we are together” attitude. | 我々の職場では、共に働こう、という姿勢がある | 我々の職場（診療科）では、共に働こう、という姿勢がある |
| 3 | People feel understood and accepted by each other. | お互いに理解し認め合っている | お互いに理解し認め合っている |
| 4 | People in the work unit cooperate in order to help develop and apply new ideas. | 同じ部署の人々は、新しいアイディアを展開・適用するために協力しあっている | 同じ部署（診療科）の人々は、新しいアイディアを展開・適用するために協力しあっている |
| 5 | Do members of the work unit build on each other’s ideas in order to achieve the best possible outcome? | 部署のメンバーは、出来るだけ最良の成果を出すために、お互いにアイディアを出し合い、活かしあっていますか | 部署（診療科）のメンバーは、出来るだけ最良の成果を出すために、お互いにアイディアを出し合い、活かしあっている |
| 6 | Our supervisor treats us with kindness and consideration. | 上司は親切心と思いやりをもって私たちに接してくれる | 上司は親切心と思いやりをもって私たちに接してくれる |
| 7 | Our supervisor shows concern for our rights as an employee. | 上司は私たちの従業員としての権利に対して理解を示してくれる | 上司は私たちの従業員としての権利に対して理解を示してくれる |
| 8 | We can trust our supervisor. | 我々の上司は信頼できる | 我々の上司は信頼できる |
